# Resource or crisis? Cognitive functioning after widowhood and why paid work status matters

**DOI:** 10.1093/geronb/gbaf234

**Published:** 2025-11-13

**Authors:** Maria Karlene Shawn I Cabaraban, Valeria Bordone, Daniela Weber

**Affiliations:** Department of Sociology, University of Vienna, Vienna, Austria; Department of Sociology, University of Vienna, Vienna, Austria; International Institute for Applied Systems Analysis (IIASA), Wittgenstein Centre for Demography and Global Human Capital (IIASA, ÖAW, University of Vienna), Laxenburg, Austria; International Institute for Applied Systems Analysis (IIASA), Wittgenstein Centre for Demography and Global Human Capital (IIASA, ÖAW, University of Vienna), Laxenburg, Austria; Health Economics and Policy Division, Vienna University of Economics and Business, Vienna, Austria; (Social Sciences Section)

**Keywords:** Memory recall, Verbal fluency, Cognition, Labor market, Marital status

## Abstract

**Objectives:**

This study investigates the extent to which the experience of widowhood is associated with within-person changes in two key dimensions of cognitive functioning: crystallized and fluid intelligence (measured as memory recall and verbal fluency, respectively). This work enriches the empirical body of knowledge by considering whether paid work status (defined as working, retirement, or homemaking) plays a protective role in gender-specific cognitive changes associated with losing a spouse.

**Methods:**

Utilizing six waves of the Survey of Health, Ageing, and Retirement in Europe (SHARE) covering 32,089 men (*N *= 97,774) and 40,821 women (*N *= 126,998) aged 50+, two-way fixed-effects regression models were estimated to compare changes in cognitive functioning between being continuously partnered versus experiencing widowhood. We considered important heterogeneities by performing sub-sample analyses by paid work status and gender.

**Results:**

Cognitive changes were associated with widowhood, albeit markedly different by gender and across paid work status. The transition to widowhood among men was associated with reduced verbal fluency only if working. Instead, widows performed more poorly, especially in terms of memory recall, but only if they were homemakers at the time of the transition.

**Discussion:**

Paid work may serve as a cognitive resource after widowhood. However, the way in which it acts depends on gender, while being retired at the time of widowhood acts as a protection for both men and women.

Cognition is a fundamental aspect of healthy and successful aging (Rowe & Kahn, 1997). While age-related decline in cognitive functioning is expected (Salthouse, 2010), the aging process is embedded in the fabric of social relationships and is, thus, not equal for all (Fingerman et al., 2020). In the cognitive aging literature, the cognitive abilities that are most commonly found to deteriorate with age and suffer from frequent stress exposure are those associated with crystallized and fluid dimensions, respectively (Rosnick et al., 2007; see also Stawski et al., 2013 for a review). Crystallized abilities involve knowledge and skills that reflect an individual’s educational and cultural experiences, whereas fluid abilities describe one’s capacity to retrieve new information for problem solving and adaptation to a changing environment (Cattell, 1971; Horn, 1989; Lindenberger, 2001). Both these dimensions of cognitive functioning have been shown to be most vulnerable to negative life events that occur more commonly in later life, such as the death of a spouse or partner (Kung, 2020; Shin et al., 2022; Zhao et al., 2021).

At the same time, it is known that the development of cognitive abilities is largely shaped by intersecting work and family spheres (Tattarini et al., 2025) and that both significantly differ between men and women in Western countries (Aisenbrey & Fasang, 2017). Specifically, women are more commonly involved in unpaid family caregiving, which, on the one hand, puts pressure on their careers (Stafford et al., 2019; Svensson et al., 2015) but, on the other hand, might cognitively “reward” them in later life (Ice et al., 2020). Overlapping work and family responsibilities have instead been found to matter less for men’s later-life cognitive functioning since men typically maintain longer, more continuous labor market participation while at the same time being less involved in family care (Bertogg & Leist, 2023; Tattarini et al., 2025). Life course trajectories of work-family patterns thus result in gendered paid work statuses in later life—whether working, retired, or homemaking—that carry forward the cognitive implications of accumulated experiences (Stafford et al., 2019) and likely bear on how older adults adjust to adverse life changes, such as the death of a partner (Wheaton, 1990). This raises the question: *To what extent do gendered paid work status differences shape cognitive changes associated with the loss of a partner in later life?*

Our study speaks to this line of inquiry by demonstrating that cognitive shortfalls—specifically, declines in memory recall and verbal fluency—following widowhood vary across paid work status at the time of loss and gender. Our main argument is that paid work status, which is inherently gendered, reflects cognitive reserve built from accumulated work and family experiences. In later life, paid work structures the availability of a social network beyond the family as well as opportunities for cognitive engagement through mental, physical, or social activities. Thus, we expect that widowhood poses harm to older adults’ cognitive functioning, but given that work-family trajectories in Europe are strongly patterned by social factors (Firat et al., 2023), the combination of paid work status and marital status likely contributes to cognitive reserve development throughout the life course and results in gender differences in patterns of cognitive decline following marital dissolution. Following this line of reasoning, we applied fixed-effects regression models on a longitudinal sample of men and women aged 50+ from the Survey of Health, Ageing, and Retirement in Europe (SHARE) to investigate the degree to which cognitive functioning changes following the experience of marital dissolution may differ across paid work status, i.e., working, retirement, or homemaking, while accounting for unobserved confounding. Our study findings extend the widowhood-cognition literature by providing insights into the role of paid work status as a resource that protects older adults from adverse cognitive changes associated with losing a spouse.

## Analytical framework and empirical evidence

### Widowhood and cognitive functioning in later life

The extant gerontological and neuroscience research focusing on widowhood mainly draws insights from a social causation perspective encompassing two dominant frameworks: (1) the marital resource model and (2) the marital crisis model.

The *marital resource* model encompasses a possible mechanism to link widowhood and cognitive functioning, presuming that the insurance pool of economic, social, and psychological support that individuals enjoy in a partnership is protective against age-related health declines (Williams et al., 2009). Within this framework, one’s partner serves as an important sociopsychological resource by connecting individuals to extended networks of family and friends, fostering meaningful interactions and shared activities that stimulate cognitive faculties in later life (Zunzunegui et al., 2003).

Another mechanism underlying the association between widowhood and cognitive functioning is derived from the *marital crisis* model, which suggests that marital disruption through divorce or widowhood can undermine an individual’s life to the extent that it compromises their health and well-being (Williams et al., 2009). Studies in neuroscience have underscored the negative effects of stress exposure on different measures of cognitive performance, including memory recall and verbal fluency (see Mikneviciute et al., 2022 for a review). While a causal link attributing cognitive deterioration to stress exposure following widowhood is yet to be clearly established in the social science literature, a wide range of studies utilizing fixed-effects approaches have documented reduced fluid (Shin et al., 2022; Wörn et al., 2020; Zhao et al., 2021) as well as crystallized cognitive abilities (Li et al., 2023; Zhao et al., 2021), and increased odds of developing dementia (Liu et al., 2020) among people who experience losing a spouse.

### Gendered patterns of paid work status and their role in the widowhood-cognition association

The above-mentioned mechanisms underlying cognitive changes associated with losing a spouse in later life may differ in their importance depending on other overlapping life domains, such as engagement in paid work. Paid work emerges as critical in this association for two main reasons. First, by structuring opportunities for building cognitive reserve throughout the life course. The *cognitive reserve model* describes late-life cognitive functioning as a product of a broad range of activities and resources across different ages and stages in the life course biography, all of which contribute to the accumulation of cognitive reserve that enables adults to retain their cognitive abilities with increasing age (Varangis & Stern, 2020). Findings from longitudinal research, ranging from studies using sequence analysis to latent growth curve models, underscore the late-life cognitive benefits of engaging in paid work continuously or for long periods (Kobayashi & Feldman, 2019; Leist et al., 2013). Second, paid work engagement matters for maintaining cognitive abilities in older ages because it affords individuals with important roles and resources that offer a range of opportunities for mental, physical, or social engagement beyond the family, including social participation and the exercise of one’s skills in the workplace (Fisher et al., 2017; Takase et al., 2024).

However, when major life transitions occur, paid work can generate competing demands and thus compromise well-being. In the case of widowhood, paid work obligations might in fact act as a burden for the bereaved individual who must navigate work demands while at the same time adjusting to their newfound role as an unmarried adult (Perrig-Chiello et al., 2016).

The role of paid work status as a resource for maintaining cognitive functioning becomes even more meaningful when situated within the gendered patterns of the adjustment to the loss of a partner. Adjustment to such a major life transition naturally necessitates resilience, i.e., an ability or readiness to adapt, the lack of which leaves those affected adapting poorly and being vulnerable to decreased health and well-being (King et al., 2019). This vulnerability manifests differently across genders. For example, Kung (2020) found that men with strong social networks are vulnerable to having their emotional problems interfere with their daily responsibilities and productivity during the widowhood adjustment period. On the contrary, changes in economic circumstances following widowhood may be particularly challenging for women in traditional marriages where their husband assumed sole responsibility for the financial management of the household (Kung, 2020; Li et al., 2023). Therefore, competing stressors—adjusting to widowhood while managing the demands of paid work engagement—may create a stressful environment that is ultimately unfavorable for one’s cognitive abilities in a gendered way.

Overall, the presented theoretical arguments and empirical findings suggest that paid work status in the older ages may either offset or exacerbate cognitive decline (as measured by tests of memory recall and verbal fluency that capture crystallized and fluid cognitive abilities, respectively) associated with the experience of widowhood. In line with the marital resource and marital conflict models, we could anticipate a decline in cognitive performance for both men and women who experienced the loss of a spouse. Yet, such a decline depends on other gender-specific cognitive engagements in which this experience is embedded. We thus expect the dual burden of paid work commitments and widowhood adjustment to be associated with negative cognitive changes, especially for men **(Hypothesis 1)**, who find themselves shouldering both paid work and household tasks, previously likely done by their wives. Alternatively, adjustment to widowhood while no longer engaged in the labor market (i.e., retired) is expected to protect men from the negative effects of widowhood on cognitive performance **(Hypothesis 2)**. In fact, for men, there will not be a double burden in such a case, while for previously working women, the financial challenges will be less demanding. Homemakers, who are likely to have had long and/or frequent career interruptions in the past, are instead expected to show lower cognitive performance following widowhood, though this pattern is anticipated only among women **(Hypothesis 3).** 

## Data and methods

### Sample selection and variables

The data were drawn from the SHARE, a cross-national, longitudinal study of non-institutionalized adults aged 50 years and older in 27 European countries and Israel (Börsch-Supan, 2019; Börsch-Supan et al., 2013). Our empirical analyses draw on data from waves 1 (collected in 2004–2005), 2 (2006–2007), 4 (2011–2012), 5 (2013), 6 (2015), and 8 (2019–2020).

**Figure 1.**
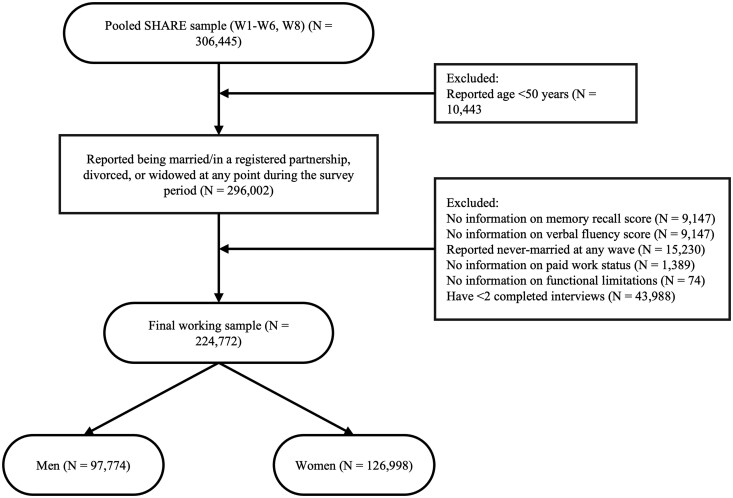
Flow diagram for the sample selection procedure. Values shown in the flow diagram represent person-year observations (N).

Figure 1 presents the flow diagram for our sample selection procedure: starting with a pooled SHARE sample of *N *= 306,445, we restricted our working sample to men and women aged 50+ (*N *= 296,002). Then, we further excluded respondents with missing information on our variables of interest, i.e., cognitive assessments (*N *= 9,147 for memory recall and *N *= 1,402 for verbal fluency), paid work status (*N *= 1,389), and functional limitations (*N *= 74). Our empirical analyses focus on individuals who were at-risk of experiencing marital dissolution (divorce or widowhood); for this reason, we excluded respondents who reported being “never-married” at any wave (*N *= 15,230). Lastly, we excluded those who did not participate in at least two consecutive SHARE waves (*N *= 43,988). Hence, the final working sample included *N* = 97,774 observations (*N *= 32,089) from men and *N *= 126,998 observations (*N *= 40,821) from women. See Supplementary Table 1 in the online Supplementary Material for sample descriptive statistics.

### Dependent variables

The analyses considered three cognitive functioning tests in SHARE to build our outcome variables: two recall tests (i.e., immediate and delayed recall) and verbal fluency. Our focus on the tests measuring recall and verbal fluency was motivated by two reasons: First, they are sensitive to cognitive aging (Stawski et al., 2013). And second, previous studies on older adults have used tests measuring recall performance to capture crystallized cognitive abilities, as well as tests measuring verbal fluency to examine fluid cognitive abilities (Salthouse, 2006; Weber et al., 2017). For the memory recall tasks in SHARE, a list of 10 words was read aloud, and respondents were asked to recall as many of these words as they could immediately afterwards (immediate recall) and after a delay without rereading the words (delayed recall). For the verbal fluency task, respondents were asked to name as many animals as possible within 60 s.

In the descriptive and multivariate analyses, the scores for immediate and delayed recall were summed to generate a summative score for memory recall, going from 0 to 20. Verbal fluency score represents the total number of animals that each participant was able to correctly produce (excluding repetitions) within the given time frame, ranging from 0 to 100.

### Independent variables

#### Widowhood status

In our analyses, widowhood is a time-varying categorical variable that distinguishes respondents’ self-reported marital status for each wave: 0 = *married/partnered* (including those in legal and consensual marriages, as well as in registered partnerships, regardless of living arrangement with their spouse or partner) serves as the reference group and 1 = *widowed* (i.e., respondent experienced the death of a spouse or partner and is not in a partnership at the time of interview). We include a third category (2 = *divorced)* to account for all possible marital transitions. It should be noted that, given the small sample of respondents transitioning from married to divorced over the observed period, the results related to the effect of such marital disruption on cognition will not be interpreted.

#### Paid work status

Information on respondents’ paid work status was derived from the Employment and Pension module of SHARE, where respondents report their employment situation for each wave. Past studies using SHARE have relied on this self-reported information in measuring employment (e.g., Mazzonna & Peracchi, 2012). Our analyses distinguished three categories: 0 = *working* serves as the reference category; 1 = *retired;* and 2 = *homemaker*, a residual category that includes homemakers as well as respondents being unemployed or sick/disabled. The label reflects its composition, with 60% (*N *= 22,086) of this residual group (*N *= 36,974) being homemakers.

### Control variables

Multivariate analyses control for a set of variables that potentially confound the association between explanatory and dependent variables, drawing on previous related literature (Bertogg & Leist, 2021; Bordone & Weber, 2013): age (continuous), wave (dummies) to consider period effects, and limitations in Activities of Daily Living (ADL) (0 = *none* [ref], 1 = *have at least 1*) as a proxy for physical health. The ADL measure provides an objective, performance-based assessment of functional capacity that captures limitations in daily functioning (e.g., difficulties in bathing, dressing, eating, toileting, and moving from one position to another), representing what individuals can and cannot do regardless of underlying pathology, unlike self-assessed measures of overall health and diagnosed illnesses, which may be subject to reporting biases and recall errors (Hajek & König, 2016). While a link between later-life depression and cognitive deterioration has been previously raised (see Koenig et al., 2014 for a more detailed discussion), the two variables may be potentially endogenous with each other (Scult et al., 2017). To avoid this methodological issue, we refrained from including depression as a control but carried out a robustness check including a lagged measure for depressive symptoms using the EURO-D scale (see section on sensitivity analyses below).

### Analytic strategy

To investigate changes in cognitive functioning associated with widowhood, we employed two-way fixed effects linear regression for panel data (Woolridge, 2003). This method examines within-person change over time in the exposure variables (marital dissolution and paid work status) to predict within-person change in the outcome variables (i.e., memory recall and verbal fluency scores). The choice of a fixed effects approach is motivated by methodological considerations. First, this statistical model uses each individual as their own control by comparing individuals’ performance in the cognitive tasks before and after they became widowed. Second, fixed effects regression accounts for unobserved time-invariant confounders that vary across individuals, such as country and educational attainment (Kohler et al., 2012). Third, the fixed effects model addresses a central limitation of cross-sectional studies in which cognitive assessments are merely compared across different sub-groups without considering changes that may occur within these sub-groups. In doing so, the fixed effects model provides estimates of cognitive changes associated with becoming widowed, while controlling for pre-existing characteristics that are associated with both the likelihood of widowhood and cognitive functioning (e.g., age).

Our multivariate analyses were conducted in several steps: First, we estimated fixed effects models in which memory recall and verbal fluency scores, respectively, were regressed on our time-varying marital dissolution variable. Next, we performed these analyses separately depending on the respondent’s paid work status. In this method of model specification, we tested whether changes in marital status (i.e., from being married to widowed) contribute to changes in cognitive performance and whether there are differences across paid work statuses in this association. A negative coefficient was interpreted as a decrease in cognitive functioning. We applied stratified models to avoid conflating changes in marital and employment status and to present subgroup-specific dynamics in a more interpretable way. Third, we assessed whether the association between our explanatory and outcome variables remained statistically significant when our controls (SHARE wave, age, and ADL limitations) were included in the model. Finally, to ascertain gender differences, we estimated separate models for men and women.

For more robust estimates, all models were adjusted for clustering at the individual-level. Results of the Hausman tests indicated that the random effects models should be abandoned in favor of the fixed effects models that use within-cluster information. All analyses were conducted using Stata 18.0.

### Sensitivity analyses

To ensure the robustness of our results, we carried out the following sensitivity analyses. First, we run the same fixed-effects models as in the main analyses on the sample of respondents who were married or partnered at the first interview (*N *= 93,606). In this way, we examine cognitive changes only for individuals who were exposed to the risk of marital dissolution. Second, to consider potential timing effects, we conducted additional analysis in which we distinguished between individuals who experienced marital dissolution at younger ages (<65 years old) versus those who experienced such an event later in life (65+). Third, additional sensitivity analyses tested whether our findings hold when we account for changes in mental health. To address potential endogeneity issues in this respect, we included a lagged variable in the main models measuring symptoms of depression on the EURO-D scale at the interview before widowhood.

## Results

### Descriptive findings

Descriptive statistics for the analytical sample by gender (Supplementary Table 1, see online supplementary material) show that more women than men are widowed (21% vs. 6%, respectively) and homemakers (24% vs. 7%), while the opposite holds for being active in the labor market (28% of men compared to 22% of women) and retired (65% of men vs. 54% of women). We observe no distinct age patterns across gender. Most individuals in the sample report having no limitations in the ADL. The distribution of respondents is about the same between men and women across survey waves (Supplementary Table 1, see online supplementary material).

Consistent with findings from previous literature (e.g., Weber et al., 2014), women generally outperformed men with respect to cognitive tests that measure memory recall (Supplementary Table 2, see online supplementary material). For both measures of cognitive functioning, unpartnered (i.e., divorced and widowed) men and women fared worse than their married/partnered counterparts. Across paid work status, the highest scores in memory recall and verbal fluency were observed in working respondents. Moreover, our descriptive findings showed that women who have formally exited from the labor force (i.e., retired) outperformed those who identified as homemakers.

More men reported having experienced marital dissolution (divorced or widowed), whereas women more commonly report being in a partnership (Supplementary Table 3, see online supplementary material), irrespective of paid work status. In Supplementary Table 4 (see online supplementary material), we report the transition percentages for the two explanatory variables. We show the percentages of individuals whose marital status remained the same or, alternatively, changed during the follow-up rounds of SHARE. Among men, about 2% of those who were married/partnered became widowed at a subsequent time; the corresponding figure for women is about 5%. For both genders, a negligible percentage transitioned from married/partnered to divorced at any point during the study period. For this reason, we only interpret findings for widows and widowers in our multivariate analyses.

Similarly, the bottom part of Supplementary Table 4 (see online supplementary material) presents the percentages of those who remained and, conversely, transitioned out of the three categories of paid work status: working, retired, and homemaker. The percentage of sample persons who transitioned out of work and into retirement is about 25% for men and 22% for women. The corresponding figures for working individuals who transitioned to other categories of paid work status are comparatively lower: about 6% for men and 9% for women. These transition percentages suggest that there is enough within-individual variability over time in our explanatory variables to argue a fixed effects approach.

### Multivariate results

**Figure 2.**
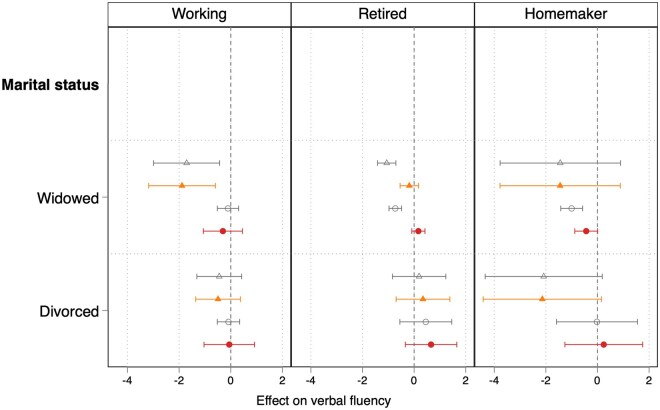
Coefficients with 95% CIs from the fixed-effects models regressing memory recall on widowhood, by gender and paid work status. Coefficients with 95% CIs are estimated from fixed-effects models regressing memory recall on widowhood for the sample of men and women aged 50+, by gender and paid work status. *Source*. Survey of Health, Ageing, and Retirement in Europe (W1, 2, 4, 5, 6, 8), release 8.0.0. Authors’ own calculations (sample weights not used). Men and women aged 50+ with at least two completed interviews and no missing information on all dependent and independent variables. See Supplementary Table 5 (see online supplementary material) for full results. Hollow triangle represents estimates for Model 1 (unadjusted model without controls) for men; solid triangle represents estimates for Model 2 (model controlling for age, wave dummies, and activities of daily living) for men; hollow circle represents estimates for Model 1 (unadjusted model without controls) for women; solid circle represents estimates for Model 2 (model controlling for age, wave dummies, and activities of daily living) for women.

**Figure 3.**
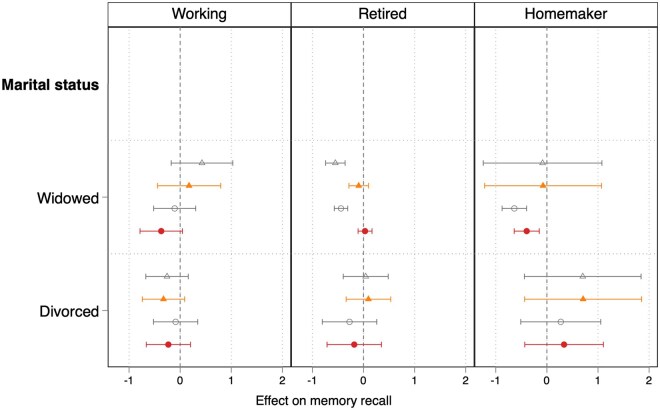
Coefficients with 95% CIs from the fixed-effects models regressing verbal fluency on widowhood, by gender and paid work status. Coefficients with 95% CIs are estimated from fixed-effects models regressing verbal fluency on widowhood for the sample of men and women aged 50+, by gender and paid work status. *Source*. Survey of Health, Ageing, and Retirement in Europe (W1, 2, 4, 5, 6, 8), release 8.0.0. Authors’ own calculations (sample weights not used). Men and women aged 50+ with at least two completed interviews and no missing information on all dependent and independent variables. See Supplementary Table 6 (see online supplementary material) for full results. Hollow triangle represents estimates for Model 1 (unadjusted model without controls) for men; solid triangle represents estimates for Model 2 (model controlling for age, wave dummies, and activities of daily living) for men; hollow circle represents estimates for Model 1 (unadjusted model without controls) for women; solid circle represents estimates for Model 2 (model controlling for age, wave dummies, and activities of daily living) for women.

Results from the fixed effects models, including the unadjusted coefficients and the full model with all the covariates entered simultaneously, are displayed in Figure 2 (for memory recall) and Figure 3 (for verbal fluency). In the unadjusted models (Model 1) that do not include controls, the expected negative coefficients for respondents at widowhood were significant only among retired widows (b = −0.55, *p *< .001) and widowers (b = −0.44, *p *< .001). These associations, however, were no longer statistically significant after controlling for age, wave dummies, and ADL (Model 2). The same happens for verbal fluency (Figure 3), apart from working men, who face a decline in their performance that is significantly associated with widowhood, even after including the controls. Homemaking women show a significant negative association between widowhood and memory recall performance in the unadjusted as well as in the full models.

### Sensitivity analyses

To test the robustness of our findings, we performed the following sensitivity analyses. First, when carrying out the main analysis on the sub-sample at risk of experiencing marital dissolution, we found qualitatively very similar results for both memory recall (Supplementary Table 7, see online supplementary material) and verbal fluency (Supplementary Table 8, see online supplementary material), suggesting that the relatively high share of respondents remaining married throughout the interview period does not affect our results.

Second, a comparison of the widowhood experience at younger (<65 years old) and older (65+) ages (Supplementary Tables 9 and 10, see online supplementary material) revealed that the negative coefficient for widowhood among working men is driven by widowers below age 65, and that also for younger working women, a similar effect exists. On the contrary, it is the older group of homemaking women who drive the negative effects of widowhood above the age of 65.

Third, we incorporated a lagged variable measuring symptoms of depression on the EURO-D scale in our fixed-effects models in order to capture the role of mental health changes. These results (Supplementary Figure 1, see online supplementary material) generally corroborate our main findings, although when controlling for changes in mental health before marital disruption, the coefficient for widowhood is no longer statistically significant for working men. While this might indicate that mental health changes constitute an important confounder in the association between widowhood and verbal fluency for men who are in paid work when facing that transition, endogeneity might still be at play: mental health changes at the wave before widowhood might, in fact, capture the effects of the causes of death. We therefore carefully interpret these results and welcome further studies on these aspects, possibly with data that contain more detailed timing information.

## Discussion

Drawing from models of marital resource and crisis, as well as cognitive reserve models, this study sought to better understand how an important role that individuals occupy over life, i.e., participation in the labor market, may be protective of cognitive functioning (in its fluid and crystallized dimensions) when experiencing the loss of a spouse. We utilized fixed-effects regressions on a longitudinal sample of men and women aged 50+ residing in Europe, focusing on whether shortfalls in these two distinct cognitive components, amounting to reductions in memory recall and verbal fluency, respectively, differ across paid work status (working, retired, homemaking) at the time of widowhood. Our findings point to reduced cognitive performance associated with widowhood, but with heterogeneities across gender and paid work status. Specifically, we found that widowhood is significantly associated with a decline in verbal fluency among men engaged in paid work, showing support for Hypothesis 1. On the contrary, confirming Hypothesis 3, widowhood is negatively associated with cognitive performance among homemaking women.

Two main themes form our contribution to the current state of the art. First, our findings contribute to a strand of literature that argues for gender differences in the cognitive health disadvantages of widowhood in later life (Li et al., 2023; Zhao et al., 2021). Our analyses are in line with previous work (e.g., Bertogg & Leist, 2021), also using SHARE data, showing that although widowed older adults exhibit reduced recall and fluency performance, this negative effect of widowhood is no longer significant once potential pathways and confounders are considered. We add to this an analysis of heterogeneities in the effects of losing a spouse between men and women, at least with respect to changes in their cognitive abilities. Marital crisis—the social and economic stressors that accompany widowhood—represents a potential mechanism for reduced cognitive performance, but it does so differently for men and women. On the one hand, men’s fluid abilities, i.e., their capacity to apply newly acquired information to adapt to a changing environment, decline. On the other hand, widows are vulnerable to a decline in the crystallized dimension of cognition (i.e., knowledge and skills obtained through educational and cultural experiences, accumulated throughout the life course).

Second, we answer questions about the role of paid work engagement in shaping cognitive changes associated with widowhood. We hypothesized that paid work status at the time of marital dissolution would play a role in whether and how widowhood reduces cognitive performance through two competing mechanisms: on the one hand, workplace cognitive stimulation may help offset cognitive decline associated with the stressful role transition to widowhood (Wheaton, 1990). On the other hand, workplace demands may create additional stress for individuals who are simultaneously restructuring their social roles to reflect their new status as an unmarried adult (Kung, 2020; Perrig-Chiello et al., 2016).

Our study shows support for Hypothesis 1 and Hypothesis 3, with widowhood being significantly associated with a decline in verbal fluency among men engaged in paid work and in memory recall among homemaking women. Hypothesis 2 was confirmed as well: the cognitive performance of widows and widowers is not significantly affected by widowhood if they are retired at the time of this role transition. This suggests that retirement, rather than employment, can have a protective effect against cognitive deterioration after marital dissolution.

Our findings align with research showing gender-specific health declines following marital dissolution. Research on widowhood has found men to suffer from mental health declines, including increased loneliness and depressive symptoms, and functional limitations in the aftermath of spousal loss (Kung, 2020; Perrig-Chiello et al., 2016; Song & Kim, 2024; Streeter, 2020). Women face similar health declines after widowhood, though these effects are more pronounced among the socioeconomically disadvantaged (Li et al., 2023; Streeter, 2020), often with elevated mortality risk (Dabergott, 2022). The present study corroborates these patterns while extending the literature by demonstrating that paid work status at the time of widowhood serves as an important moderator, structuring exposure to cognitive demands and stressors in gender-specific ways during the widowhood adjustment period.

We also acknowledge some limitations of this study. First, our measure of widowhood relied on self-reporting of marital status. While this is typical in investigations on widowhood and cognitive functioning (e.g., Shin et al., 2022; Wörn et al., 2020), respondents might have already formed a new partnership at the time of interview but still consider themselves as widowed. Moreover, individuals (particularly men) who experienced a transition to widowhood in the sample analyzed do not constitute a very large portion of the total sample, resulting often in large CIs of the estimates. Similarly, paid work status fundamentally relies on individuals’ subjective interpretation of what constitutes “retirement” or being out of the labor force. In this sense, this approach may potentially result in a misclassification of (especially women’s) paid work status. Second, although the fixed-effects model accounts for individual characteristics before *and* after the onset of widowhood, the possibility of health selection into widowhood cannot be completely ruled out. A third limitation of our study is that the role of social support following the widowhood could not be considered, as such analyses would have had to rely on further reduced sample sizes, resulting in less robust estimates. Future research is invited to develop a novel approach to examine marital status changes, paid work status, support from family members and friends, and cognition simultaneously; the importance of social support may be particularly relevant for widows, for whom the effect of such support likely buffers the cognitive disadvantages of losing a spouse or partner (Lee & Jiang, 2023). Fourth, health changes following widowhood may attenuate with increasing duration. For example, Wörn et al. (2020) observed a decline in reasoning ability among women only in the second year after widowhood onset, after which cognitive changes were no longer significant. In our case, information on the year of the spouse’s death was, however, missing for 45.5% of the sample, and removing those cases would have significantly biased our sample. Yet, considering the relatively short intervals in between the survey years, we do not expect potential overestimation to be severe enough to bias our results.

Despite these limitations, this study adds to previous longitudinal studies in answering questions about cognitive shortfalls associated with marital dissolution. Our results point to a gendered role of paid work in the association between widowhood and cognitive decline. These findings open a broader discussion on formulating more nuanced policies that help mitigate cognitive decline among widowed older adults by providing enhanced survivor benefits, flexible work arrangements, and gender-specific bereavement support programs integrated within existing European Union social protection frameworks.

## Supplementary Material

gbaf234_Supplementary_Data

## Data Availability

Registered researchers can download waves 1, 2, 4, 5, 6, and 8 of the Survey of Health, Ageing and Retirement in Europe from the SHARE Research Data Center (doi: https://doi.org/10.6103/SHARE.w4.800; https://doi.org/10.6103/SHARE.w6.800). For more information, visit https://share-eric.eu/data/data-access.
