# Supplementary material for: Resource or crisis? Cognitive functioning after widowhood and why paid work status matters
Source: J Gerontol B Psychol Sci Soc Sci. 2025 Nov 13;81(1):gbaf234. doi: 10.1093/geronb/gbaf234 (PMC12771523; doi:10.1093/geronb/gbaf234)
Supplement: gbaf234_Supplementary_Data [file gbaf234_supplementary_data.zip › JGSS suppl Cabaraban, Bordone, & Weber.docx]

***The Journals of Gerontology, Series B: Psychological Sciences and Social Sciences* Supplementary Material: Cabaraban, Bordone, & Weber. Resource or crisis? Cognitive functioning after widowhood and why paid work status matters.**

**Supplementary Table 1**. *Frequency and Percentage (in Parentheses) Distribution of Study Variables*

|  | **Men** | **Women** | **Total sample** | **p-value** |
| --- | --- | --- | --- | --- |
| **N =** | **97,774** | **126,998** | **N = 224,772** |  |
| **n =** | **32,089** | **40,821** | **72,910** |  |
| **Marital status** |  |  |  |  |
| Married/Partnered | 84,374 (86.3%) | 87,133 (68.6%) | 171,507 (76.3%) | <0.001 |
| Widowed | 6,228 (6.4%) | 27,083 (21.3%) | 33,311 (14.8%) |  |
| Divorced | 7,172 (7.3%) | 12,782 (10.1%) | 19,954 (8.9%) |  |
| **Paid work status** |  |  |  |  |
| Working | 27,353 (28.0%) | 28,490 (22.4%) | 55,843 (24.8%) | <0.001 |
| Retired | 63,739 (65.2%) | 68,216 (53.7%) | 131,955 (58.7%) |  |
| Homemaker | 6,682 (6.8%) | 30,292 (23.9%) | 36,974 (16.4%) |  |
| **Age in 5-year groups** |  |  |  |  |
| 50-54 | 7,560 (7.7%) | 12,101 (9.5%) | 19,661 (8.7%) | <0.001 |
| 55-59 | 14,779 (15.1%) | 20,701 (16.3%) | 35,480 (15.8%) |  |
| 60-64 | 18,216 (18.6%) | 23,296 (18.3%) | 41,512 (18.5%) |  |
| 65-69 | 18,659 (19.1%) | 22,543 (17.8%) | 41,202 (18.3%) |  |
| 70-74 | 15,719 (16.1%) | 18,861 (14.9%) | 34,580 (15.4%) |  |
| 75-79 | 12,005 (12.3%) | 14,925 (11.8%) | 26,930 (12.0%) |  |
| 80+ | 10,836 (11.1%) | 14,571 (11.5%) | 25,407 (11.3%) |  |
| **Age (Mean ± SD)** | 67.25 ± (9.09) | 66.83 ± (9.42) | 67.01 ± (9.28) | <0.001 |
| **Have at least 1 ADL difficulty** |  |  |  |  |
| None | 89,278 (91.3%) | 113,955 (89.7%) | 203,233 (90.4%) | <0.001 |
| Have at least 1 | 8,496 (8.7%) | 13,043 (10.3%) | 21,539 (9.6%) |  |
| **[Wave] Survey year** |  |  |  |  |
| [W1] 2004-05 | 9,105 (9.3%) | 11,062 (8.7%) | 20,167 (9.0%) | <0.001 |
| [W2] 2006-07 | 12,072 (12.3%) | 14,906 (11.7%) | 26,978 (12.0%) |  |
| [W4] 2011-12 | 18,143 (18.6%) | 24,056 (18.9%) | 42,199 (18.8%) |  |
| [W5] 2013 | 22,411 (22.9%) | 28,968 (22.8%) | 51,379 (22.9%) |  |
| [W6] 2015 | 22,581 (23.1%) | 29,697 (23.4%) | 52,278 (23.3%) |  |
| [W8] 2019-20 | 13,462 (13.8%) | 18,309 (14.4%) | 31,771 (14.1%) |  |

*Notes.* p-values are based on Chi-square tests of independence (categorical) and t-test (continuous). Source: SHARE (W1, 2, 4, 5, 6, 8), release 8.0.0. Authors’ own calculations (sample weights not used).

**Supplementary Table 2**. *Mean Cognition Scores Among the Sample Adults Aged 50+*

| **Characteristics** | **Memory recall** | | | | | **Verbal fluency** | | | | |
| --- | --- | --- | --- | --- | --- | --- | --- | --- | --- | --- |
|  | **Males** | | **Females** | | **Sig** | **Males** | | **Females** | | **Sig** |
|  | **N** | ***M***  ***(SD)*** | **N** | ***M***  ***(SD)*** |  | **N** | ***M***  ***(SD)*** | **N** | ***M***  ***(SD)*** |  |
| Total sample | 97,774 | 8.85 (1.91) | 126,998 | 9.43 (1.96) | *** | 97,774 | 20.41 (4.04) | 126,998 | 20.17 (3.87) | *** |
| *Marital status* |  |  |  |  |  |  |  |  |  |  |
| Married/ Partnered | 84,374 | 8.90 (1.90) | 87,133 | 9.74 (1.93) | *** | 84,374 | 20.47 (4.02) | 87,133 | 20.65 (3.81) | *** |
| Widowed | 6,228 | 7.57 (1.72) | 27,083 | 7.96 (1.84) | *** | 6,228 | 18.18 (3.60) | 27,083 | 17.67 (3.52) | *** |
| Divorced | 7,172 | 9.43 (1.86) | 12,782 | 10.38 (1.95) | *** | 7,172 | 21.67 (3.90) | 12,782 | 22.23 (4.09) | *** |
| *Paid work status* |  |  |  |  |  |  |  |  |  |  |
| Working | 27,353 | 10.26 (1.67) | 28,490 | 11.32 (1.72) | *** | 27,353 | 22.88 (3.62) | 28,490 | 23.78 (3.55) | *** |
| Retired | 63,739 | 8.25 (1.82) | 68,216 | 9.04 (1.86) | *** | 63,739 | 19.42 (3.75) | 68,216 | 19.83 (3.63) | *** |
| Homemaker | 6,682 | 8.79 (1.42) | 30,292 | 8.52 (1.70) | *** | 6,682 | 19.69 (3.15) | 30,292 | 17.54 (3.29) | *** |

*Notes.* N = number of observations; *M* = Mean; SD = Standard deviation (within-person); **** p* < 0.001. Source: SHARE (W1, 2, 4, 5, 6, 8), release 8.0.0. Authors’ own calculations (sample weights not used).

**Supplementary Table 3**. *Frequency and Percentage (in Parentheses) Distribution of Marital Status Across Categories of Paid Work Status*

| Marital status | Paid work status | | | | | |
| --- | --- | --- | --- | --- | --- | --- |
|  | Working | | Retired | | Homemaker | |
|  | Males | Females | Males | Females | Males | Females |
| Married/Partnered | 22,359 | 24,187 | 41,765 | 54,700 | 23,009 | 5,487 |
|  | (78.48) | (88.43) | (61.22) | (85.82) | (75.96) | (82.12) |
| Widowed | 1,889 | 550 | 19,626 | 5,484 | 5,568 | 194 |
|  | (6.63) | (2.01) | (28.77) | (8.60) | (18.38) | (2.90) |
| Divorced | 4,242 | 2,616 | 6,825 | 3,555 | 1,715 | 1,001 |
|  | (14.89) | (9.56) | (10.00) | (5.58) | (5.66) | (14.98) |
| Total | 28,490 | 27,353 | 68,216 | 63,739 | 30,292 | 6,682 |
|  | (100.00) | (100.00) | (100.00) | (100.00) | (100.00) | (100.00) |

*Notes.* Source: SHARE (W1, 2, 4, 5, 6, 8), release 8.0.0. Authors’ own calculations (sample weights not used).

**Supplementary Table 4**. *Transitions in Marital Status and Paid Work Status Among the Sample Men and Women Aged 50+, By Gender*

|  | Marital transitions | | | |
| --- | --- | --- | --- | --- |
|  | Married/Partnered | Widowed | Divorced | Total |
| *Males* |  |  |  |  |
| Married/Partnered | 55,934 | 1,134 | 261 | 57,329 |
|  | (97.57) | (1.98) | (0.46) | (100.00) |
| Widowed | 53 | 3,514 | 5 | 3,572 |
|  | (1.48) | (98.38) | (0.14) | (100.00) |
| Divorced | 175 | 23 | 4,586 | 4,784 |
|  | (3.66) | (0.48) | (95.86) | (100.00) |
| *Females* |  |  |  |  |
| Married/Partnered | 57,372 | 3,203 | 269 | 60.844 |
|  | (94.29) | (5.26) | (0.44) | (100.00) |
| Widowed | 45 | 16,537 | 19 | 16,601 |
|  | (0.27) | (99.61) | (0.11) | (100.00) |
| Divorced | 156 | 45 | 8,531 | 8,732 |
|  | (1.79) | (0.52) | (10.23) | (100.00) |
|  | Paid work status transitions | | | |
|  | Working | Retired | Other |  |
| *Males* |  |  |  |  |
| Working | 14,444 | 5,355 | 1,228 | 21,027 |
|  | (68.69) | (25.47) | (5.84) | (100.00) |
| Retired | 444 | 38,791 | 568 | 39,803 |
|  | (1.12) | (97.46) | (1.43) | (100.00) |
| Homemaker | 662 | 2,056 | 2,137 | 4,855 |
|  | (13.64) | (42.35) | (44.02) | (100.00) |
| *Females* |  |  |  |  |
| Working | 15,167 | 4,714 | 1,887 | 21,768 |
|  | (69.68) | (21.66) | (8.67) | (100.00) |
| Retired | 364 | 40,297 | 2,347 | 43,008 |
|  | (0.85) | (93.70) | (5.46) | (100.00) |
| Homemaker | 1,144 | 5,265 | 14,992 | 21,401 |
|  | (5.35) | (24.60) | (70.05) | (100.00) |

*Notes.* First row shows frequencies and second row shows row percentages (in parentheses).Source: SHARE (W1, 2, 4, 5, 6, 8), release 8.0.0. Authors’ own calculations (sample weights not used).

**Supplementary Table 5**. *Coefficients and 95% Confidence Intervals from Fixed Effects (FE) Regressions Estimating the Association between Marital Dissolution and Memory Recall Among the Sample Men and Women Aged 50+, By Gender and Paid Work Status (Full Results)*

|  | Working | | | | Retired | | | | Homemaker | | | |
| --- | --- | --- | --- | --- | --- | --- | --- | --- | --- | --- | --- | --- |
|  | Men | | Women | | Men | | Women | | Men | | Women | |
| Memory recall | Model 1 | Model 2 | Model 1 | Model 2 | Model 1 | Model 2 | Model 1 | Model 2 | Model 1 | Model 2 | Model 1 | Model 2 |
| **Marital dissolution** |  |  |  |  |  |  |  |  |  |  |  |  |
| Married/ Partnered | Ref | Ref | Ref | Ref | Ref | Ref | Ref | Ref | Ref | Ref | Ref | Ref |
| Widowed | 0.43 | 0.17 | -0.11 | -0.37 | -0.55^***^ | -0.09 | -0.44^***^ | 0.03 | -0.08 | -0.07 | -0.64^***^ | -0.39^**^ |
|  | [-0.18,1.03] | [-0.44,0.79] | [-0.52,0.30] | [-0.79,0.04] | [-0.74,-0.36] | [-0.29,0.10] | [-0.57,-0.31] | [-0.11,0.17] | [-1.25,1.08] | [-1.22,1.07] | [-0.88,-0.40] | [-0.64,-0.15] |
| Divorced | -0.26 | -0.33 | -0.09 | -0.23 | 0.04 | 0.10 | -0.27 | -0.18 | 0.70 | 0.71 | 0.27 | 0.34 |
|  | [-0.67,0.16] | [-0.74,0.09] | [-0.52,0.34] | [-0.66,0.20] | [-0.40,0.48] | [-0.34,0.53] | [-0.81,0.26] | [-0.71,0.35] | [-0.44,1.84] | [-0.43,1.85] | [-0.51,1.05] | [-0.43,1.11] |
| **Covariates** |  |  |  |  |  |  |  |  |  |  |  |  |
| Age |  | -0.02 |  | 0.08 |  | -0.02 |  | -0.03 |  | -0.12 |  | -0.11^*^ |
|  |  | [-0.12,0.07] |  | [-0.02,0.18] |  | [-0.09,0.05] |  | [-0.09,0.04] |  | [-0.36,0.11] |  | [-0.21,-0.01] |
| *SHARE wave* |  |  |  |  |  |  |  |  |  |  |  |  |
| 1 |  | Ref |  | Ref |  | Ref |  | Ref |  | Ref |  | Ref |
| 2 |  | 0.24 |  | 0.14 |  | 0.11 |  | 0.18 |  | 0.58 |  | 0.43^**^ |
|  |  | [-0.03,0.52] |  | [-0.16,0.43] |  | [-0.08,0.31] |  | [-0.01,0.37] |  | [-0.13,1.28] |  | [0.15,0.72] |
| 4 |  | 0.66^*^ |  | 0.12 |  | 0.05 |  | -0.13 |  | 1.16 |  | 0.80^*^ |
|  |  | [0.01,1.30] |  | [-0.57,0.81] |  | [-0.42,0.51] |  | [-0.58,0.32] |  | [-0.46,2.77] |  | [0.12,1.49] |
| 5 |  | 0.87^*^ |  | 0.16 |  | -0.05 |  | -0.12 |  | 1.54 |  | 0.90^*^ |
|  |  | [0.05,1.70] |  | [-0.72,1.04] |  | [-0.65,0.55] |  | [-0.70,0.46] |  | [-0.50,3.58] |  | [0.02,1.78] |
| 6 |  | 1.01^*^ |  | 0.16 |  | -0.12 |  | -0.12 |  | 1.77 |  | 1.10^*^ |
|  |  | [0.00,2.02] |  | [-0.92,1.24] |  | [-0.85,0.62] |  | [-0.83,0.58] |  | [-0.73,4.27] |  | [0.02,2.17] |
| 8 |  | 1.00 |  | -0.34 |  | -0.61 |  | -0.55 |  | 2.08 |  | 1.23 |
|  |  | [-0.44,2.43] |  | [-1.88,1.20] |  | [-1.65,0.43] |  | [-1.55,0.45] |  | [-1.46,5.62] |  | [-0.30,2.76] |
| *ADL limitations* |  |  |  |  |  |  |  |  |  |  |  |  |
| None |  | Ref |  | Ref |  | Ref |  | Ref |  | Ref |  | Ref |
| Have at least 1 |  | -0.21 |  | -0.07 |  | -0.51^***^ |  | -0.32^***^ |  | -0.25 |  | -0.32^***^ |
|  |  | [-0.49,0.06] |  | [-0.32,0.18] |  | [-0.60,-0.41] |  | [-0.41,-0.22] |  | [-0.60,0.11] |  | [-0.47,-0.17] |
| Constant | 10.28^***^ | 11.00^***^ | 11.34^***^ | 6.72^**^ | 8.30^***^ | 10.05^***^ | 9.19^***^ | 11.31^***^ | 8.68^***^ | 14.89^*^ | 8.62^***^ | 15.02^***^ |
|  | [10.24,10.32] | [6.17,15.83] | [11.27,11.41] | [1.71,11.73] | [8.27,8.33] | [5.73,14.38] | [9.13,9.26] | [7.18,15.44] | [8.50,8.87] | [2.66,27.11] | [8.56,8.68] | [9.21,20.83] |
| N | 27353 | 27353 | 28490 | 28490 | 63739 | 63739 | 68216 | 68216 | 6682 | 6682 | 30292 | 30292 |
| *R*^2^ | 0.00 | 0.01 | 0.00 | 0.01 | 0.00 | 0.02 | 0.00 | 0.02 | 0.00 | 0.01 | 0.00 | 0.01 |
| Adjusted *R*^2^ | 0.00 | 0.01 | -0.00 | 0.01 | 0.00 | 0.02 | 0.00 | 0.02 | 0.00 | 0.00 | 0.00 | 0.01 |
| *AIC* | 105667 | 105458 | 111725 | 111430 | 257263 | 255963 | 278256 | 277166 | 23620 | 23604 | 117895 | 117715 |
| *BIC* | 105684 | 105532 | 111741 | 111505 | 257281 | 256044 | 278274 | 277248 | 23634 | 23666 | 117912 | 117790 |
| RMSE | 1.67 | 1.66 | 1.72 | 1.71 | 1.82 | 1.80 | 1.86 | 1.85 | 1.42 | 1.41 | 1.69 | 1.69 |

*Notes.* Model 1 does not include controls, Model 2 controls for age, age-squared, number of ADL limitations, and SHARE interview years; 95% confidence intervals in brackets; AIC = Akaike Information Criteria; BIC = Bayesian Information Criteria; RMSE = Root Mean Squared Errors; ^*^ *p* < 0.05, ^**^ *p* < 0.01, ^***^ *p* < 0.001. Source: SHARE (W1, 2, 4, 5, 6, 8), release 8.0.0.

**Supplementary Table 6**. *Coefficients and 95% Confidence Intervals from Fixed Effects (FE) Regressions Estimating the Association between Marital Dissolution and Verbal Fluency Among the Sample Men and Women Aged 50+, By Gender and Paid Work Status (Full Results)*

|  | Working | | | | Retired | | | | Homemaker | | | |
| --- | --- | --- | --- | --- | --- | --- | --- | --- | --- | --- | --- | --- |
|  | Men | | Women | | Men | | Women | | Men | | Women | |
| Verbal fluency | Model 1 | Model 2 | Model 1 | Model 2 | Model 1 | Model 2 | Model 1 | Model 2 | Model 1 | Model 2 | Model 1 | Model 2 |
| **Marital dissolution** |  |  |  |  |  |  |  |  |  |  |  |  |
| Married/ Partnered | Ref | Ref | Ref | Ref | Ref | Ref | Ref | Ref | Ref | Ref | Ref | Ref |
| Widowed | -1.71^**^ | -1.89^**^ | 0.10 | -0.30 | -1.06^***^ | -0.19 | -0.73^***^ | 0.17 | -1.44 | -1.45 | -1.00^***^ | -0.44 |
|  | [-2.99,-0.43] | [-3.18,-0.60] | [-0.65,0.84] | [-1.06,0.45] | [-1.42,-0.71] | [-0.54,0.17] | [-0.97,-0.49] | [-0.09,0.42] | [-3.77,0.89] | [-3.78,0.88] | [-1.42,-0.58] | [-0.88,0.01] |
| Divorced | -0.45 | -0.49 | 0.08 | -0.06 | 0.20 | 0.34 | 0.45 | 0.66 | -2.08 | -2.14 | -0.02 | 0.24 |
|  | [-1.31,0.42] | [-1.36,0.37] | [-0.90,1.05] | [-1.04,0.92] | [-0.84,1.23] | [-0.70,1.39] | [-0.55,1.46] | [-0.34,1.65] | [-4.35,0.18] | [-4.43,0.15] | [-1.59,1.55] | [-1.26,1.75] |
| **Covariates** |  |  |  |  |  |  |  |  |  |  |  |  |
| Age |  | 0.19 |  | 0.14 |  | -0.08 |  | -0.15^*^ |  | 0.32 |  | 0.27^**^ |
|  |  | [-0.04,0.41] |  | [-0.07,0.35] |  | [-0.22,0.06] |  | [-0.27,-0.03] |  | [-0.19,0.83] |  | [0.07,0.47] |
| *SHARE wave* |  |  |  |  |  |  |  |  |  |  |  |  |
| 1 |  | Ref |  | Ref |  | Ref |  | Ref |  | Ref |  | Ref |
| 2 |  | -0.46 |  | -0.12 |  | -0.29 |  | -0.37^*^ |  | -0.85 |  | -0.62^*^ |
|  |  | [-1.12,0.19] |  | [-0.73,0.48] |  | [-0.68,0.10] |  | [-0.73,-0.01] |  | [-2.25,0.55] |  | [-1.19,-0.05] |
| 4 |  | -1.68^*^ |  | -1.10 |  | -1.07^*^ |  | -0.40 |  | -2.53 |  | -2.71^***^ |
|  |  | [-3.23,-0.12] |  | [-2.51,0.32] |  | [-2.02,-0.12] |  | [-1.22,0.43] |  | [-6.02,0.96] |  | [-4.08,-1.34] |
| 5 |  | -1.71 |  | -0.97 |  | -0.93 |  | -0.25 |  | -3.18 |  | -3.31^***^ |
|  |  | [-3.70,0.28] |  | [-2.79,0.84] |  | [-2.15,0.29] |  | [-1.30,0.80] |  | [-7.57,1.21] |  | [-5.05,-1.56] |
| 6 |  | -1.85 |  | -0.96 |  | -0.97 |  | -0.07 |  | -3.62 |  | -3.96^***^ |
|  |  | [-4.29,0.59] |  | [-3.19,1.27] |  | [-2.47,0.52] |  | [-1.36,1.22] |  | [-9.05,1.81] |  | [-6.12,-1.80] |
| 8 |  | -2.21 |  | -1.26 |  | -1.18 |  | -0.16 |  | -4.78 |  | -4.94^**^ |
|  |  | [-5.69,1.26] |  | [-4.44,1.92] |  | [-3.29,0.92] |  | [-1.99,1.66] |  | [-12.57,3.01] |  | [-8.00,-1.88] |
| *ADL limitations* |  |  |  |  |  |  |  |  |  |  |  |  |
| None |  | Ref |  | Ref |  | Ref |  | Ref |  | Ref |  | Ref |
| Have at least 1 |  | -1.12^***^ |  | -0.39 |  | -1.31^***^ |  | -0.78^***^ |  | -0.37 |  | -1.00^***^ |
|  |  | [-1.70,-0.55] |  | [-0.91,0.13] |  | [-1.50,-1.11] |  | [-0.95,-0.61] |  | [-1.18,0.43] |  | [-1.29,-0.70] |
| Constant | 22.96^***^ | 13.45^*^ | 23.77^***^ | 16.44^**^ | 19.50^***^ | 25.99^***^ | 19.99^***^ | 30.77^***^ | 20.04^***^ | 3.50 | 17.73^***^ | 2.50 |
|  | [22.87,23.05] | [1.85,25.06] | [23.61,23.92] | [6.01,26.88] | [19.44,19.57] | [17.24,34.73] | [19.87,20.12] | [23.24,38.30] | [19.69,20.39] | [-23.57,30.57] | [17.61,17.85] | [-9.07,14.07] |
| N | 27353 | 27353 | 28490 | 28490 | 63739 | 63739 | 68216 | 68216 | 6682 | 6682 | 30292 | 30292 |
| *R*^2^ | 0.00 | 0.01 | 0.00 | 0.00 | 0.00 | 0.02 | 0.00 | 0.02 | 0.00 | 0.00 | 0.00 | 0.01 |
| Adjusted *R*^2^ | 0.00 | 0.00 | -0.00 | 0.00 | 0.00 | 0.02 | 0.00 | 0.02 | 0.00 | 0.00 | 0.00 | 0.01 |
| *AIC* | 148008 | 147893 | 153065 | 152945 | 349257 | 347890 | 369257 | 368180 | 34291 | 34291 | 158157 | 157785 |
| *BIC* | 148025 | 147967 | 153082 | 153019 | 349275 | 347971 | 369275 | 368262 | 34305 | 34352 | 158174 | 157860 |
| RMSE | 3.62 | 3.61 | 3.55 | 3.54 | 3.75 | 3.71 | 3.62 | 3.60 | 3.15 | 3.15 | 3.29 | 3.27 |

*Notes.* Model 1 does not include controls, Model 2 controls for age, age-squared, number of ADL limitations, and SHARE interview years; 95% confidence intervals in brackets; AIC = Akaike Information Criteria; BIC = Bayesian Information Criteria; RMSE = Root Mean Squared Errors; ^*^ *p* < 0.05, ^**^ *p* < 0.01, ^***^ *p* < 0.001. Source: SHARE (W1, 2, 4, 5, 6, 8), release 8.0.0.

**Supplementary Table 7**. *Coefficients and 95% Confidence Intervals from Fixed-effects Regressions Estimating the Association Between Widowhood and Memory Recall Among Married/Partnered Individuals Aged 50+ at t_1_ Among the Sample Men and Women Aged 50+, By Gender and Paid Work Status (Full Results)*

|  | Working | | | | Retired | | | | Others | | | |
| --- | --- | --- | --- | --- | --- | --- | --- | --- | --- | --- | --- | --- |
|  | Males | | Females | | Males | | Females | | Males | | Females | |
| Memory recall | Unadjusted | Full | Unadjusted | Full | Unadjusted | Full | Unadjusted | Full | Unadjusted | Full | Unadjusted | Full |
| **Marital dissolution** |  |  |  |  |  |  |  |  |  |  |  |  |
| Married/ Partnered | Ref | Ref | Ref | Ref | Ref | Ref | Ref | Ref | Ref | Ref | Ref | Ref |
| Widowed | 0.36 | 0.08 | -0.08 | -0.36 | -0.60^***^ | -0.13 | -0.46^***^ | 0.01 | -0.09 | -0.06 | -0.66^***^ | -0.42^**^ |
|  | [-0.23,0.95] | [-0.52,0.69] | [-0.51,0.35] | [-0.80,0.08] | [-0.79,-0.40] | [-0.32,0.07] | [-0.59,-0.33] | [-0.13,0.15] | [-1.56,1.39] | [-1.53,1.41] | [-0.90,-0.42] | [-0.67,-0.17] |
| Divorced | -0.05 | -0.26 | 0.10 | -0.23 | -0.07 | 0.32 | -0.19 | 0.21 | 1.42^*^ | 1.40^*^ | 0.42 | 0.61 |
|  | [-0.60,0.50] | [-0.82,0.29] | [-0.41,0.60] | [-0.74,0.28] | [-0.67,0.53] | [-0.27,0.91] | [-0.89,0.50] | [-0.48,0.90] | [0.17,2.66] | [0.13,2.67] | [-0.56,1.40] | [-0.37,1.58] |
| **Covariates** |  |  |  |  |  |  |  |  |  |  |  |  |
| Age |  | -0.02 |  | 0.08 |  | -0.04 |  | -0.01 |  | 0.01 |  | -0.09 |
|  |  | [-0.11,0.08] |  | [-0.03,0.19] |  | [-0.11,0.03] |  | [-0.09,0.07] |  | [-0.24,0.27] |  | [-0.21,0.02] |
| *SHARE Wave* |  |  |  |  |  |  |  |  |  |  |  |  |
| 1 |  | Ref |  | Ref |  | Ref |  | Ref |  | Ref |  | Ref |
| 2 |  | 0.24 |  | 0.15 |  | 0.19 |  | 0.07 |  | 0.04 |  | 0.44^**^ |
|  |  | [-0.05,0.53] |  | [-0.18,0.48] |  | [-0.01,0.40] |  | [-0.18,0.31] |  | [-0.75,0.83] |  | [0.12,0.77] |
| 4 |  | 0.62 |  | 0.10 |  | 0.20 |  | -0.39 |  | 0.05 |  | 0.74 |
|  |  | [-0.06,1.29] |  | [-0.68,0.87] |  | [-0.30,0.69] |  | [-0.96,0.18] |  | [-1.73,1.83] |  | [-0.04,1.52] |
| 5 |  | 0.80 |  | 0.13 |  | 0.13 |  | -0.41 |  | 0.16 |  | 0.76 |
|  |  | [-0.06,1.66] |  | [-0.85,1.12] |  | [-0.50,0.77] |  | [-1.14,0.31] |  | [-2.08,2.40] |  | [-0.23,1.75] |
| 6 |  | 0.94 |  | 0.12 |  | 0.11 |  | -0.42 |  | 0.17 |  | 0.91 |
|  |  | [-0.12,1.99] |  | [-1.09,1.33] |  | [-0.67,0.89] |  | [-1.30,0.47] |  | [-2.57,2.92] |  | [-0.30,2.13] |
| 8 |  | 0.87 |  | -0.37 |  | -0.27 |  | -0.89 |  | -0.16 |  | 1.02 |
|  |  | [-0.62,2.37] |  | [-2.09,1.36] |  | [-1.37,0.83] |  | [-2.13,0.36] |  | [-4.05,3.73] |  | [-0.71,2.75] |
| *ADL difficulties* |  |  |  |  |  |  |  |  |  |  |  |  |
| None |  | Ref |  | Ref |  | Ref |  | Ref |  | Ref |  | Ref |
| Have at least 1 |  | -0.18 |  | -0.10 |  | -0.51^***^ |  | -0.36^***^ |  | -0.24 |  | -0.29^***^ |
|  |  | [-0.48,0.12] |  | [-0.39,0.18] |  | [-0.61,-0.40] |  | [-0.48,-0.24] |  | [-0.65,0.16] |  | [-0.47,-0.12] |
| Constant | 10.24^***^ | 10.53^***^ | 11.32^***^ | 6.58^*^ | 8.31^***^ | 11.24^***^ | 9.38^***^ | 10.64^***^ | 8.75^***^ | 7.90 | 8.74^***^ | 14.09^***^ |
|  | [10.23,10.24] | [5.49,15.56] | [11.31,11.32] | [1.00,12.16] | [8.30,8.31] | [6.67,15.81] | [9.37,9.39] | [5.64,15.64] | [8.74,8.77] | [-5.53,21.33] | [8.73,8.75] | [7.67,20.52] |
| N | 24318 | 24318 | 22752 | 22752 | 56257 | 56257 | 45506 | 45506 | 5535 | 5535 | 24184 | 24184 |
| *R*^2^ | 0.00 | 0.01 | 0.00 | 0.01 | 0.00 | 0.02 | 0.00 | 0.02 | 0.00 | 0.00 | 0.00 | 0.01 |
| Adjusted *R*^2^ | 0.00 | 0.01 | -0.00 | 0.01 | 0.00 | 0.02 | 0.00 | 0.02 | 0.00 | 0.00 | 0.00 | 0.01 |
| *AIC* | 93998 | 93816 | 89356 | 89139 | 227128 | 226045 | 184897 | 184166 | 19317 | 19315 | 95207 | 95071 |
| *BIC* | 94014 | 93889 | 89372 | 89211 | 227146 | 226125 | 184914 | 184245 | 19330 | 19375 | 95223 | 95143 |
| RMSE | 1.67 | 1.67 | 1.72 | 1.72 | 1.82 | 1.80 | 1.85 | 1.83 | 1.39 | 1.38 | 1.73 | 1.73 |

*Notes.* 95% confidence intervals in brackets; AIC = Akaike Information Criteria; BIC = Bayesian Information Criteria; RMSE = Root Mean Squared Errors; ^*^ *p* < 0.05, ^**^ *p* < 0.01, ^***^ *p* < 0.001. Source: SHARE (W1, 2, 4, 5, 6, 8), release 8.0.0.

**Supplementary Table 8**. *Coefficients and 95% Confidence Intervals from Fixed-effects Regression Models Estimating the Association Between Widowhood and Verbal Fluency Among Married/Partnered Men and Women Aged 50+ at t_1_, By Gender and Paid Work Status (Full Results)*

|  | Working | | | | Retired | | | | Others | | | |
| --- | --- | --- | --- | --- | --- | --- | --- | --- | --- | --- | --- | --- |
|  | Males | | Females | | Males | | Females | | Males | | Females | |
| Verbal fluency | Model 1 | Model 2 | Model 1 | Model 2 | Model 1 | Model 2 | Model 1 | Model 2 | Model 1 | Model 2 | Model 1 | Model 2 |
| **Marital dissolution** |  |  |  |  |  |  |  |  |  |  |  |  |
| Married/ Partnered | Ref | Ref | Ref | Ref | Ref | Ref | Ref | Ref | Ref | Ref | Ref | Ref |
| Widowed | -2.39^***^ | -2.64^***^ | 0.22 | -0.25 | -1.18^***^ | -0.29 | -0.75^***^ | 0.07 | -0.85 | -0.95 | -1.03^***^ | -0.51^*^ |
|  | [-3.71,-1.07] | [-3.97,-1.31] | [-0.57,1.01] | [-1.06,0.56] | [-1.54,-0.82] | [-0.66,0.07] | [-0.99,-0.50] | [-0.19,0.33] | [-3.59,1.89] | [-3.71,1.81] | [-1.45,-0.60] | [-0.96,-0.06] |
| Divorced | -0.55 | -0.90 | -0.19 | -0.63 | -0.57 | 0.06 | -0.77 | -0.09 | -2.98 | -3.28^*^ | 0.11 | 0.49 |
|  | [-1.80,0.70] | [-2.15,0.34] | [-1.51,1.14] | [-1.95,0.70] | [-2.08,0.93] | [-1.48,1.59] | [-1.93,0.40] | [-1.26,1.08] | [-6.17,0.21] | [-6.50,-0.06] | [-1.51,1.72] | [-1.07,2.04] |
| **Covariates** |  |  |  |  |  |  |  |  |  |  |  |  |
| Age |  | 0.18 |  | 0.07 |  | -0.04 |  | -0.09 |  | 0.49 |  | 0.36^**^ |
|  |  | [-0.06,0.41] |  | [-0.16,0.30] |  | [-0.19,0.11] |  | [-0.24,0.06] |  | [-0.08,1.05] |  | [0.14,0.58] |
| *SHARE Wave* |  |  |  |  |  |  |  |  |  |  |  |  |
| 1 |  | Ref |  | Ref |  | Ref |  | Ref |  | Ref |  | Ref |
| 2 |  | -0.35 |  | 0.13 |  | -0.31 |  | -0.49^*^ |  | -1.57^*^ |  | -0.75^*^ |
|  |  | [-1.04,0.33] |  | [-0.53,0.79] |  | [-0.74,0.11] |  | [-0.95,-0.04] |  | [-3.13,-0.01] |  | [-1.40,-0.11] |
| 4 |  | -1.54 |  | -0.57 |  | -1.23^*^ |  | -0.85 |  | -3.93^*^ |  | -3.19^***^ |
|  |  | [-3.16,0.09] |  | [-2.12,0.98] |  | [-2.25,-0.20] |  | [-1.89,0.20] |  | [-7.83,-0.03] |  | [-4.72,-1.66] |
| 5 |  | -1.56 |  | -0.26 |  | -1.15 |  | -0.65 |  | -4.70 |  | -3.96^***^ |
|  |  | [-3.64,0.51] |  | [-2.25,1.73] |  | [-2.46,0.16] |  | [-1.97,0.68] |  | [-9.59,0.20] |  | [-5.90,-2.02] |
| 6 |  | -1.64 |  | -0.14 |  | -1.24 |  | -0.59 |  | -5.46 |  | -4.77^***^ |
|  |  | [-4.19,0.90] |  | [-2.58,2.31] |  | [-2.85,0.36] |  | [-2.21,1.03] |  | [-11.51,0.59] |  | [-7.18,-2.36] |
| 8 |  | -1.92 |  | -0.02 |  | -1.56 |  | -0.92 |  | -7.39 |  | -6.07^***^ |
|  |  | [-5.55,1.70] |  | [-3.50,3.46] |  | [-3.82,0.70] |  | [-3.22,1.38] |  | [-16.05,1.27] |  | [-9.48,-2.66] |
| *ADL difficulties* |  |  |  |  |  |  |  |  |  |  |  |  |
| None |  | Ref |  | Ref |  | Ref |  | Ref |  | Ref |  | Ref |
| Have at least 1 |  | -1.12^***^ |  | -0.36 |  | -1.33^***^ |  | -0.75^***^ |  | -0.40 |  | -0.97^***^ |
|  |  | [-1.74,-0.51] |  | [-0.95,0.24] |  | [-1.54,-1.12] |  | [-0.97,-0.52] |  | [-1.34,0.54] |  | [-1.30,-0.64] |
| Constant | 22.82^***^ | 13.84^*^ | 23.76^***^ | 19.90^***^ | 19.50^***^ | 23.74^***^ | 20.39^***^ | 27.48^***^ | 19.63^***^ | -5.59 | 17.84^***^ | -1.89 |
|  | [22.81,22.83] | [1.71,25.97] | [23.75,23.78] | [8.52,31.28] | [19.49,19.51] | [14.39,33.10] | [20.37,20.41] | [18.23,36.72] | [19.59,19.66] | [-35.72,24.54] | [17.82,17.86] | [-14.53,10.74] |
| N | 24318 | 24318 | 22752 | 22752 | 56257 | 56257 | 45506 | 45506 | 5535 | 5535 | 24184 | 24184 |
| *R*^2^ | 0.00 | 0.01 | 0.00 | 0.01 | 0.00 | 0.02 | 0.00 | 0.01 | 0.00 | 0.01 | 0.00 | 0.01 |
| Adjusted *R*^2^ | 0.00 | 0.01 | -0.00 | 0.00 | 0.00 | 0.02 | 0.00 | 0.01 | 0.00 | 0.00 | 0.00 | 0.01 |
| *AIC* | 131891 | 131787 | 122286 | 122184 | 308423 | 307305 | 245429 | 244828 | 28227 | 28221 | 126759 | 126472 |
| *BIC* | 131907 | 131860 | 122302 | 122257 | 308441 | 307386 | 245446 | 244906 | 28240 | 28280 | 126775 | 126545 |
| RMSE | 3.64 | 3.63 | 3.56 | 3.55 | 3.75 | 3.71 | 3.59 | 3.56 | 3.10 | 3.09 | 3.33 | 3.31 |

*Notes.* 95% confidence intervals in brackets; AIC = Akaike Information Criteria; BIC = Bayesian Information Criteria; RMSE = Root Mean Squared Errors; ^*^ *p* < 0.05, ^**^ *p* < 0.01, ^***^ *p* < 0.001. Source: SHARE (W1, 2, 4, 5, 6, 8), release 8.0.0.

**Supplementary Table 9**. *Coefficients and 95% Confidence Intervals from Fixed-effects Regression Models Estimating the Association Between Widowhood and Memory Recall Among Sample Men and Women Aged 50+, By Gender, Paid Work Status, and Age Group (Full Results)*

|  | Working | | | | Retired | | | | Others | | | |
| --- | --- | --- | --- | --- | --- | --- | --- | --- | --- | --- | --- | --- |
|  | Males | | Females | | Males | | Females | | Males | | Females | |
| Memory recall | aged <65 | aged 65+ | aged <65 | aged 65+ | aged <65 | aged 65+ | aged <65 | aged 65+ | aged <65 | aged 65+ | aged <65 | aged 65+ |
| **Marital dissolution** |  |  |  |  |  |  |  |  |  |  |  |  |
| Married/ Partnered | Ref | Ref | Ref | Ref | Ref | Ref | Ref | Ref | Ref | Ref | Ref | Ref |
| Widowed | 0.06 | 0.10 | -0.50^*^ | 0.60 | -0.00 | -0.02 | 0.02 | 0.07 | 0.13 | -1.28^*^ | 0.35 | -0.31^*^ |
|  | [-0.71,0.83] | [-1.03,1.24] | [-0.97,-0.04] | [-0.81,2.01] | [-1.29,1.28] | [-0.21,0.18] | [-0.73,0.77] | [-0.08,0.22] | [-1.29,1.55] | [-2.47,-0.08] | [-0.17,0.87] | [-0.61,-0.01] |
| Divorced | -0.20 | 0.41 | -0.21 | -0.19 | 0.03 | 0.21 | -1.04 | -0.38 | 0.78 | -2.86^***^ | 0.12 | 0.38 |
|  | [-0.64,0.24] | [-1.89,2.71] | [-0.66,0.23] | [-1.94,1.55] | [-1.05,1.12] | [-0.33,0.76] | [-2.82,0.74] | [-1.02,0.26] | [-0.43,1.99] | [-4.34,-1.38] | [-0.67,0.92] | [-3.47,4.23] |
| **Covariates** |  |  |  |  |  |  |  |  |  |  |  |  |
| Age | 0.04 | -0.43^*^ | 0.10 | -0.16 | -0.03 | -0.07 | 0.11 | -0.10^*^ | -0.08 | -0.45 | -0.03 | -0.18^*^ |
|  | [-0.07,0.15] | [-0.86,-0.01] | [-0.01,0.21] | [-0.63,0.32] | [-0.27,0.22] | [-0.15,0.00] | [-0.10,0.31] | [-0.17,-0.02] | [-0.36,0.20] | [-1.36,0.46] | [-0.17,0.12] | [-0.34,-0.02] |
| *SHARE Wave* |  |  |  |  |  |  |  |  |  |  |  |  |
| 1 | Ref | Ref | Ref | Ref | Ref | Ref | Ref | Ref | Ref | Ref | Ref | Ref |
| 2 | 0.11 | 1.16 | 0.12 | -0.14 | 0.42 | 0.19 | 0.20 | 0.26^*^ | 0.53 | -0.33 | 0.37 | 0.43 |
|  | [-0.21,0.42] | [-0.28,2.61] | [-0.20,0.44] | [-2.03,1.75] | [-0.20,1.04] | [-0.03,0.40] | [-0.37,0.76] | [0.04,0.48] | [-0.27,1.34] | [-4.00,3.33] | [-0.05,0.78] | [-0.03,0.88] |
| 4 | 0.29 | 2.55 | 0.05 | 0.37 | 0.78 | 0.20 | -0.13 | 0.10 | 0.93 | 1.31 | 0.65 | 0.82 |
|  | [-0.47,1.06] | [-0.04,5.15] | [-0.71,0.80] | [-2.95,3.69] | [-0.85,2.41] | [-0.32,0.72] | [-1.54,1.27] | [-0.43,0.63] | [-1.01,2.87] | [-4.38,6.99] | [-0.37,1.66] | [-0.26,1.91] |
| 5 | 0.39 | 3.77^*^ | 0.06 | 0.83 | 0.97 | 0.14 | -0.16 | 0.16 | 1.30 | 1.63 | 0.61 | 1.02 |
|  | [-0.59,1.37] | [0.45,7.10] | [-0.91,1.02] | [-3.29,4.94] | [-1.13,3.06] | [-0.52,0.81] | [-1.94,1.62] | [-0.51,0.84] | [-1.17,3.77] | [-5.44,8.70] | [-0.69,1.91] | [-0.37,2.41] |
| 6 | 0.46 | 4.41^*^ | 0.02 | 1.01 | 1.05 | 0.15 | -0.11 | 0.23 | 1.46 | 2.18 | 0.79 | 1.22 |
|  | [-0.74,1.65] | [0.23,8.59] | [-1.16,1.20] | [-4.02,6.03] | [-1.52,3.62] | [-0.67,0.96] | [-2.28,2.06] | [-0.60,1.06] | [-1.56,4.48] | [-6.73,11.09] | [-0.80,2.38] | [-0.48,2.92] |
| 8 | 0.24 | 6.03^*^ | -0.56 | 1.36 | 1.09 | -0.19 | -0.70 | 0.02 | 1.66 | 4.65 | 0.89 | 1.43 |
|  | [-1.45,1.94] | [0.01,12.05] | [-2.23,1.12] | [-5.76,8.48] | [-2.56,4.74] | [-1.34,0.96] | [-3.76,2.36] | [-1.15,1.18] | [-2.62,5.94] | [-8.40,17.70] | [-1.37,3.16] | [-1.00,3.86] |
| *ADL difficulties* |  |  |  |  |  |  |  |  |  |  |  |  |
| None | Ref | Ref | Ref | Ref | Ref | Ref | Ref | Ref | Ref | Ref | Ref | Ref |
| Have at least 1 | -0.29 | 0.26 | -0.01 | -1.20 | -0.36 | -0.51^***^ | -0.27 | -0.28^***^ | -0.26 | -0.68 | -0.15 | -0.23^*^ |
|  | [-0.59,0.02] | [-0.74,1.26] | [-0.27,0.25] | [-2.44,0.05] | [-0.78,0.06] | [-0.61,-0.40] | [-0.60,0.07] | [-0.38,-0.17] | [-0.64,0.13] | [-2.02,0.67] | [-0.39,0.10] | [-0.43,-0.04] |
|  |  |  |  |  |  |  |  |  |  |  |  |  |
| Constant | 7.82^**^ | 35.54^**^ | 5.69^*^ | 20.51 | 10.31 | 13.40^***^ | 4.08 | 15.88^***^ | 12.28 | 37.71 | 10.59^**^ | 19.67^***^ |
|  | [2.24,13.39] | [10.10,60.97] | [0.32,11.06] | [-8.07,49.09] | [-2.83,23.45] | [8.48,18.32] | [-6.78,14.93] | [10.89,20.86] | [-2.05,26.61] | [-19.57,95.00] | [2.99,18.19] | [9.14,30.20] |
| N | 24491 | 2862 | 26545 | 1945 | 10289 | 53450 | 12764 | 55452 | 5775 | 907 | 16789 | 13503 |
| *R*^2^ | 0.01 | 0.02 | 0.01 | 0.01 | 0.01 | 0.03 | 0.01 | 0.03 | 0.01 | 0.08 | 0.00 | 0.03 |
| Adjusted *R*^2^ | 0.01 | 0.01 | 0.01 | 0.01 | 0.01 | 0.03 | 0.01 | 0.03 | 0.01 | 0.07 | 0.00 | 0.03 |
| *AIC* | 93542 | 8951 | 103297 | 6130 | 33303 | 212001 | 43234 | 222539 | 20455 | 2244 | 63116 | 50142 |
| *BIC* | 93615 | 9005 | 103371 | 6180 | 33368 | 212081 | 43302 | 222619 | 20515 | 2283 | 63185 | 50210 |
| RMSE | 1.63 | 1.15 | 1.69 | 1.17 | 1.22 | 1.76 | 1.32 | 1.80 | 1.42 | 0.83 | 1.58 | 1.55 |

*Notes*. 95% confidence intervals in brackets; AIC = Akaike Information Criteria; BIC = Bayesian Information Criteria; RMSE = Root Mean Squared Errors; ^*^ *p* < 0.05, ^**^ *p* < 0.01, ^***^ *p* < 0.001. Source: SHARE (W1, 2, 4, 5, 6, 8), release 8.0.0.**Supplementary Table 10**. *Coefficients and 95% Confidence Intervals from Fixed-effects Regression Models Estimating the Association Between Widowhood and Verbal Fluency Among the Sample Men and Women Aged 50+, By Gender, Paid Work Status, and Age Group (Full Results)*

|  | Working | | | | Retired | | | | Others | | | | |
| --- | --- | --- | --- | --- | --- | --- | --- | --- | --- | --- | --- | --- | --- |
|  | Males | | Females | | Males | | Females | | Males | | Females | | |
| Verbal fluency | aged <65 | aged 65+ | aged <65 | aged 65+ | aged <65 | aged 65+ | aged <65 | aged 65+ | aged <65 | aged 65+ | aged <65 | aged 65+ |  |
| **Marital dissolution** | |  |  |  |  |  |  |  |  |  |  |  |  |
| Married/ Partnered | Ref | Ref | Ref | Ref | Ref | Ref | Ref | Ref | Ref | Ref | Ref | Ref |  |
| Widowed | -2.11^**^ | -1.81 | -0.18 | -2.05 | 1.92 | -0.23 | 0.87 | 0.20 | -1.79 | 1.92 | 0.14 | -0.25 |  |
|  | [-3.55,-0.66] | [-6.28,2.65] | [-1.01,0.65] | [-5.75,1.65] | [-0.65,4.49] | [-0.60,0.14] | [-0.53,2.26] | [-0.07,0.47] | [-5.03,1.45] | [-0.60,4.44] | [-0.80,1.07] | [-0.84,0.34] |  |
| Divorced | -0.39 | -7.02^**^ | -0.17 | -3.10 | 0.04 | 0.67 | 0.15 | 0.45 | -2.17 | -1.17 | 0.40 | -0.41 |  |
|  | [-1.33,0.54] | [-11.50,-2.54] | [-1.16,0.82] | [-9.29,3.08] | [-3.49,3.56] | [-0.54,1.87] | [-2.30,2.60] | [-0.79,1.70] | [-4.59,0.24] | [-4.18,1.84] | [-1.38,2.19] | [-2.74,1.92] |  |
| **Covariates** |  |  |  |  |  |  |  |  |  |  |  |  |  |
| Age | 0.12 | 0.08 | 0.21 | -0.06 | 0.32 | -0.13 | 0.27 | -0.23^***^ | 0.30 | -1.29 | 0.40^**^ | 0.11 |  |
|  | [-0.14,0.38] | [-0.84,1.01] | [-0.02,0.43] | [-0.93,0.82] | [-0.29,0.93] | [-0.28,0.01] | [-0.20,0.75] | [-0.37,-0.10] | [-0.29,0.89] | [-3.34,0.76] | [0.10,0.70] | [-0.17,0.38] |  |
| *SHARE Wave* |  |  |  |  |  |  |  |  |  |  |  |  |  |
| 1 | Ref | Ref | Ref | Ref | Ref | Ref | Ref | Ref | Ref | Ref | Ref | Ref |  |
| 2 | -0.22 | -0.68 | -0.29 | 0.32 | -0.62 | -0.28 | -0.99 | -0.24 | -0.86 | 4.32 | -0.58 | -0.67 |  |
|  | [-0.95,0.51] | [-3.65,2.30] | [-0.93,0.36] | [-2.90,3.55] | [-2.19,0.95] | [-0.70,0.14] | [-2.27,0.29] | [-0.64,0.16] | [-2.41,0.69] | [-3.60,12.24] | [-1.42,0.27] | [-1.44,0.11] |  |
| 4 | -1.04 | -3.02 | -1.49 | -1.79 | -2.63 | -1.00 | -1.89 | -0.28 | -2.29 | 6.72 | -2.81^**^ | -2.39^*^ |  |
|  | [-2.82,0.74] | [-9.29,3.25] | [-3.02,0.04] | [-7.51,3.92] | [-6.65,1.38] | [-2.02,0.03] | [-5.17,1.40] | [-1.19,0.63] | [-6.29,1.71] | [-7.32,20.76] | [-4.86,-0.75] | [-4.22,-0.55] |  |
| 5 | -0.94 | -2.46 | -1.45 | -2.01 | -2.68 | -0.80 | -2.24 | -0.10 | -2.87 | 8.21 | -3.71^**^ | -2.60^*^ |  |
|  | [-3.22,1.34] | [-10.48,5.56] | [-3.42,0.51] | [-9.39,5.37] | [-7.91,2.56] | [-2.11,0.51] | [-6.41,1.94] | [-1.26,1.07] | [-7.95,2.20] | [-9.06,25.49] | [-6.35,-1.08] | [-4.94,-0.26] |  |
| 6 | -0.81 | -3.03 | -1.57 | -1.64 | -3.35 | -0.77 | -2.83 | 0.19 | -3.33 | 12.42 | -4.38^**^ | -3.17^*^ |  |
|  | [-3.61,1.98] | [-12.93,6.87] | [-3.98,0.84] | [-10.77,7.49] | [-9.78,3.08] | [-2.37,0.84] | [-7.92,2.26] | [-1.24,1.62] | [-9.56,2.89] | [-9.48,34.33] | [-7.61,-1.15] | [-6.06,-0.27] |  |
| 8 | -0.53 | -4.61 | -2.03 | -1.51 | -3.66 | -0.93 | -4.00 | 0.24 | -4.38 | 17.75 | -5.52^*^ | -3.88 |  |
|  | [-4.52,3.46] | [-18.52,9.31] | [-5.47,1.41] | [-14.59,11.56] | [-12.81,5.49] | [-3.19,1.33] | [-11.19,3.20] | [-1.78,2.26] | [-13.33,4.58] | [-13.30,48.79] | [-10.13,-0.91] | [-7.98,0.22] |  |
| *ADL difficulties* | |  |  |  |  |  |  |  |  |  |  |  |  |
| None | Ref | Ref | Ref | Ref | Ref | Ref | Ref | Ref | Ref | Ref | Ref | Ref |  |
| Have at least 1 | -1.15^***^ | -0.13 | -0.37 | -0.58 | -1.50^***^ | -1.31^***^ | -0.15 | -0.76^***^ | -0.43 | -1.55 | -0.41 | -1.26^***^ |  |
|  | [-1.78,-0.53] | [-1.96,1.69] | [-0.93,0.18] | [-2.61,1.45] | [-2.29,-0.71] | [-1.52,-1.10] | [-0.82,0.53] | [-0.95,-0.57] | [-1.32,0.47] | [-4.44,1.35] | [-0.83,0.01] | [-1.69,-0.83] |  |
|  |  |  |  |  |  |  |  |  |  |  |  |  |  |
| Constant | 17.04^*^ | 19.93 | 13.37^*^ | 29.24 | 3.62 | 29.84^***^ | 7.28 | 36.50^***^ | 5.35 | 98.51 | -0.88 | 10.07 |  |
|  | [4.02,30.05] | [-35.16,75.03] | [2.23,24.50] | [-22.85,81.32] | [-29.57,36.81] | [20.20,39.48] | [-18.14,32.70] | [27.89,45.11] | [-24.83,35.54] | [-28.63,225.65] | [-16.20,14.44] | [-7.99,28.13] |  |
| N | 24491 | 2862 | 26545 | 1945 | 10289 | 53450 | 12764 | 55452 | 5775 | 907 | 16789 | 13503 |  |
| *R*^2^ | 0.01 | 0.04 | 0.01 | 0.02 | 0.01 | 0.03 | 0.00 | 0.03 | 0.00 | 0.04 | 0.01 | 0.03 |  |
| Adjusted *R*^2^ | 0.01 | 0.04 | 0.01 | 0.02 | 0.01 | 0.03 | 0.00 | 0.03 | 0.00 | 0.03 | 0.01 | 0.03 |  |
| *AIC* | 131763 | 12786 | 141897 | 8688 | 48628 | 287875 | 62319 | 293592 | 29870 | 3628 | 84635 | 69095 |  |
| *BIC* | 131836 | 12840 | 141971 | 8738 | 48693 | 287955 | 62386 | 293672 | 29930 | 3667 | 84705 | 69163 |  |
| RMSE | 3.56 | 2.26 | 3.50 | 2.25 | 2.57 | 3.57 | 2.78 | 3.42 | 3.21 | 1.78 | 3.01 | 3.12 |  |

*Notes.* 95% confidence intervals in brackets; AIC = Akaike Information Criteria; BIC = Bayesian Information Criteria; RMSE = Root Mean Squared Errors; ^*^ *p* < 0.05, ^**^ *p* < 0.01, ^***^ *p* < 0.001. Source: SHARE (W1, 2, 4, 5, 6, 8), release 8.0.0.


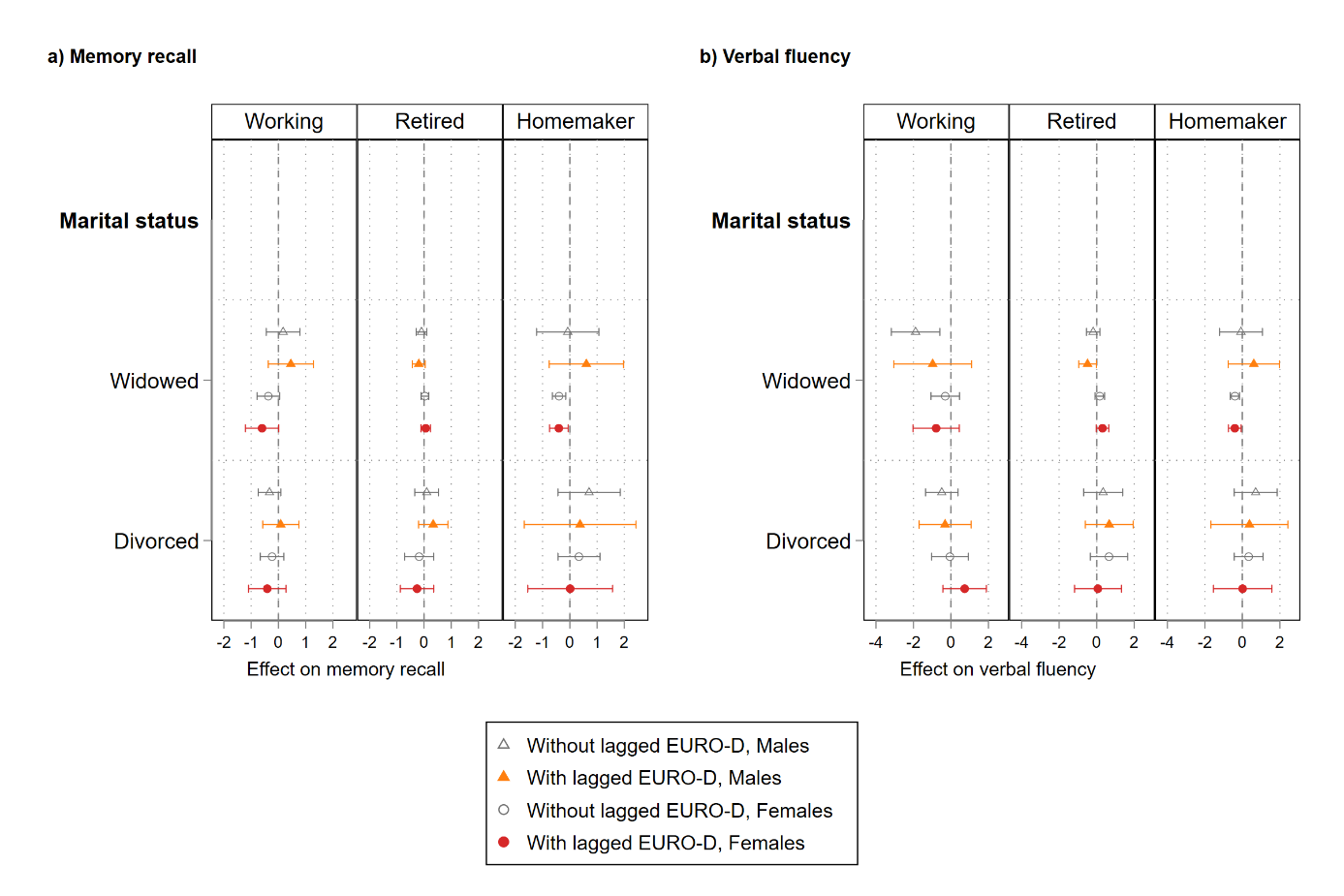


**Supplementary Figure 1**

*Coefficients with 95% Confidence Intervals from Fixed-effects Models Regressing Cognitive Measures on Widowhood and Other Covariates*

*Notes.* Coefficients with 95% confidence intervals are estimated from fixed-effects models regressing cognitive measures (Panel A for memory recall and Panel B for verbal fluency) on widowhood and other covariates for the sample men and women aged 50+, by gender and paid work status. Hollow gray markers show estimates from models without controlling for EURO-D. Solid markers show estimates from models incorporating lagged EURO-D score1. Source: SHARE (W1, 2, 4, 5, 6, 8), release 8.0.0. Authors’ own calculations (sample weights not used). Men and women aged 50+ with at least two completed interviews and no missing information on all dependent and independent variables.
